# A new method for assessing lung tumor motion in radiotherapy using dynamic chest radiography

**DOI:** 10.1002/acm2.13736

**Published:** 2022-08-05

**Authors:** Kazushi Kitamura, Kenji Takayama, Ryo Yamazaki, Yukihiro Ueda, Shigeo Nishiki

**Affiliations:** ^1^ Department of Radiology Tenri Hospital Tenri Japan; ^2^ Department of Radiation Oncology Tenri Hospital Tenri Japan; ^3^ Japanese Society of Radiological Technology Shimogyo‐ku Japan

**Keywords:** dynamic chest radiography, Kinovea, lung tumor, motion assessment, stereotactic body radiation therapy

## Abstract

Dynamic chest radiography (DCR) is a recent advanced modality to acquire dynamic and functional images. We developed a new method using DCR and the free analysis software, Kinovea, to assess lung tumor motion. This study aimed to demonstrate the usefulness of our method. Phantom and clinical studies were performed. In the phantom study, dynamic images of a moving lead sphere were acquired using DCR, and the motion of the phantom was tracked using Kinovea in a DCR video. The amplitude of phantom motion was measured and compared with a predetermined baseline amplitude. In a clinical study, DCR and respiratory‐gated four‐dimensional computed tomography (4D‐CT) were performed on 15 patients who underwent stereotactic body radiation therapy for lung tumors. The amplitudes of tumor motion in DCR and 4D‐CT were measured in the superior‐inferior (SI), left‐right (LR), and anterior‐posterior (AP) directions, and the square root of the sum of squares (SRSS) of the amplitude was calculated in all directions. Spearman's rank correlation and the Wilcoxon signed‐rank test were performed to determine the correlations of the amplitudes of tumor motion obtained using DCR and 4D‐CT. In the phantom study, the absolute mean error between the measured and predetermined amplitudes was 0.60 mm (range: 0.061.53 mm). In the clinical study, the amplitudes of tumor motion obtained using DCR correlated significantly with those of 4D‐CT in the SI and LR directions, as did the SRSS values. The median amplitudes for DCR were significantly higher than those for 4D‐CT in all (SI, LR, and AP) directions, as were the SRSS values. Our proposed method based on DCR and Kinovea is useful for assessing lung tumor motion, visually and quantitatively. Therefore, DCR has potential as a new modality for evaluating lung tumor motion in radiotherapy.

## 1 INTRODUCTION

In stereotactic body radiation therapy (SBRT) for lung tumors, it is important to minimize the effects of respiratory motion in order to precisely locate the tumor and spare healthy tissue.^1^, ^2^, ^3^, ^4^ The methods used to reduce motion effects include respiratory gating, breath‐hold, forced shallow breathing, and real‐time tumor‐tracking techniques. The method used for treatment should be decided based on tumor respiratory motion during treatment planning.

Respiratory‐gated four‐dimensional computed tomography (4D‐CT) is the primary modality used to evaluate tumor respiratory motion. 4D‐CT is useful because it affords good tumor visibility and allows measurement of three‐dimensional motion. However, 4D‐CT is prone to motion artifacts and can image only a single respiratory phase; thus, it cannot evaluate changes in the respiratory pattern over time.^5^, ^6^, ^7^, ^8^

Fluoroscopy is used to complement 4D‐CT. The American Association of Physicists in Medicine (AAPM) Task Group 76 recommends methods such as fluoroscopy to evaluate tumor respiratory motion during the initial clinical assessment.^1^ A typical SBRT workflow is as follows: (1) Fluoroscopy is performed to determine whether or not to use some methods to account for respiratory motion such as breath‐hold irradiation or breath‐synchronized irradiation. (2) CT simulation is conducted. 4D‐CT enables determination of three‐dimensional internal‐target volume by means of a treatment‐planning system. The planning‐target volume is derived from the internal‐target volume with consideration of setup error. (3) At the beginning of image‐guided radiotherapy in each session, cone‐beam CT or on‐board imaging is performed to confirm that the target lies within the planning‐target volume.

X‐ray simulators are used for fluoroscopy in many radiotherapy departments.^9^ However, such fluoroscopy can be problematic. First, lung tumors are often undetectable because of poor image quality. Second, respiratory motion is visually assessed; thus, it is subjective and lacks quantifiability.

Dynamic chest radiography (DCR) using a flat panel detector (FPD) is a recently introduced advanced dynamic X‐ray imaging modality performed in a manner similar to conventional radiography.^10^, ^11^ Dynamic FPDs were developed for cardiac imaging in the 2000s and have proven to be more useful and dose‐efficient than traditional image‐intensifier systems.^12^ Tanaka et al. developed DCR as a functional imaging method for diaphragmatic motion, pulmonary ventilation, and circulation.^13^, ^14^, ^15^ Currently, DCR systems are being used in clinical institutes to acquire high‐quality dynamic and functional images with a low radiation dose.^16^, ^17^ DCR has proven to be a simple and efficient procedure.

Here, we developed a method based on DCR and free analysis software to assess tumor motion objectively and quantitatively. Our method solves the problems associated with conventional fluoroscopy using an X‐ray simulator; it complements 4D‐CT. The aim of this study was to demonstrate the usefulness of our method for evaluating lung tumor motion in radiotherapy by conducting phantom and clinical studies.

## 2 METHODS AND MATERIALS

### 2.1 Dynamic chest radiography imaging

The DCR imaging system (Regius CS7‐H; Konica Minolta Inc., Tokyo, Japan) used in this study comprised a pulsed‐X‐ray generator (RADspeed Pro, SHIMADZU Corporation, Kyoto, Japan) and FPDs on standing and lying mounts (AeroDR, Konica Minolta Inc., Tokyo, Japan). The patient was exposed to 15 pulses per second (s), in the standing, sitting, and lying positions, over a 20‐s acquisition period for a total of 300 dynamic images during a single scan. The matrix size was 1062 × 1062 pixels, the pixel size was 400 × 400 µm,^2^ and the gray‐scale images were 16‐bit. The signal intensity and image contrast were determined automatically by the imaging system. Acquired images were imported to a KINOSIS workstation (Konica Minolta Inc., Tokyo, Japan), and then analyzed and exported to an image‐file server or external storage device.

### 2.2 The developed method

Our method consists of the following steps. First, DCR images of moving objects, such as phantoms and lung tumors in breathing patients, are acquired. Second, the DCR images are exported from the workstation as Digital Imaging and Communications in Medicine data to a stand‐alone computer running Excel (Microsoft Corporation, Redmond, WA, USA), ImageJ 1.53e (NIH, Bethesda, MD, USA),^18^ and Kinovea 0.8.27 software. Kinovea is a free, open‐source tool (available for download at http://www.kinovea.org) that is used for low‐cost kinematic analysis in sports and in patients with neurological disorders, and for tracking movement during surgery, etc.^19^, ^20^, ^21^, ^22^ Third, the DCR images obtained during a single scan are converted into an 8‐bit video file using ImageJ, and motion tracking is performed using Kinovea. During the motion‐tracking process, the object of interest is tracked automatically; its trajectory is displayed as a colored line. In Kinovea, motion tracking involves computation of the cross‐correlation coefficient between a “candidate window” and a “feature window.”^23^ Variation in the position of the object is measured, and a comma‐separated values file is exported. The amplitude of an object's motion is calculated via Excel as the peak‐to‐peak distance, after correcting for geometric magnification of the image.

### 2.3 Phantom study

**FIGURE 1:**
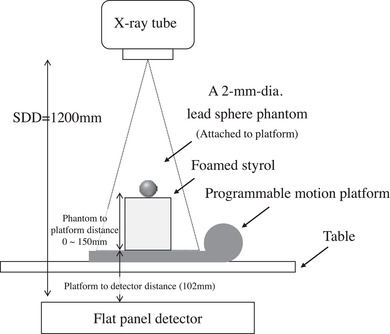
Experimental setup for the phantom study. SDD, source‐to‐detector distance

A 2‐mm‐diameter lead sphere phantom moved according to predetermined patterns of sinusoidal motion set by a programmable motion platform (QUASAR Respiratory Motion Phantom; Modus Medical Devices Inc., Ontario, Canada). The parameters of the predetermined motion patterns were as follows: amplitude, 10, 20, 30, or 40 mm; period, 2, 4, or 6 s; direction of movement, vertical only or diagonal (vertical‐horizontal with the platform rotated at 30° or 45°); orbit, linear or parallelogram; and phantom‐to‐platform distance, 0, 50, 100, or 150 mm (the corresponding geometric magnification of the phantom in the image was 1.09×, 1.15×, 1.20×, and 1.27×, respectively) (Figure 1).

DCR imaging of the phantom was performed. The X‐ray exposure conditions were as follows: tube voltage, 85 kV; tube current, 80 mA; X‐ray pulse duration, 1.0 ms; and source‐to‐detector distance, 120 cm. An 0.2‐mm copper filter was also used.

Motion tracking of the phantom was performed in each DCR video, and the amplitude of phantom motion was evaluated in the horizontal and vertical directions. The error was determined as the difference between the calculated amplitude and a predetermined baseline amplitude.

### 2.4 Clinical study

#### 2.4.1 Patient characteristics

We obtained approval from the Research Ethics Committee of Tenri Hospital (Nara, Japan) for this clinical study as part of a comprehensive program of research aimed at the development and evaluation of minimally invasive modalities for analyzing dynamic respiratory functions.

Fifteen patients who had undergone SBRT for lung tumors between December 2019 and June 2021 were included in the study. One patient underwent SBRT for two tumors simultaneously; thus, in total 16 tumors were treated. The median age of the patients was 80 years (range: 58–88 years). The tumor location in the lung was the upper lobes in six cases, middle lobe in one case, and lower lobes in nine cases. The mean tumor size was 19.2 mm (range: 7.0–39.0 mm). In all cases, the tumor observed in CT images was a solid tumor that was defined as a pure solid tumor or ground‐glass opacity (GGO) with solid parts having a consolidated maximum tumor diameter to tumor diameter ratio (C/T ratio) of more than 0.5.^24^ No fiducial markers were implanted in the patients. All patients underwent DCR imaging and 4D‐CT within 1 week (Table 1).

**TABLE 1:** Patient characteristics

Patient				Lung tumor		
Number	Age	Sex	Stage	LR side	Lobe	Size (mm)
1a	80	F	IA3	R	Lower	21.6
1b					Lower	9.7
2	67	M	IA2	R	Lower	13.0
3	82	M	IA2	L	Upper	15.0
4	88	F	IB	R	Lower	23.0
5	68	M	IVA	R	Upper	39.0
6	84	M	IA3	L	Lower	25.2
7	68	M	IA1	R	Middle	9.0
8	75	M	IVA	R	Upper	9.0
9	84	M	IB	R	Lower	32.0
10	86	M	IA3	L	Upper	25.0
11	85	M	IA2	L	Lower	16.0
12	58	M	IA1	R	Lower	7.0
13	75	M	IA3	R	Lower	25.0
14	78	F	IA3	L	Upper	23.0
15	80	M	IA2	L	Upper	16.0

#### 2.4.2 Image acquisition

DCR imaging was performed on patients in the supine position, with both arms raised during four to six free‐breathing cycles for anterior‐posterior (AP) and lateral (Lat) projections. The X‐ray exposure conditions for the AP/Lat projections were as follows: tube voltage, 85/120 kV; tube current, 80/100 mA; pulse duration, 4.0/8.0 ms; source‐to‐detector distance, 120/200 cm; and entrance surface dose, 5.0/6.4 mGy. In the Lat position, the X‐rays were projected from the healthy side to the affected (tumor) side. A DCR video included about four to six respiratory cycles during a 20‐s image‐acquisition time.

The 4D‐CT scans were performed using a 64‐slice CT scanner (LightSpeed VCT; GE Healthcare, Waukesha, WI, USA) during free breathing in cine mode, with respiratory monitoring performed using a Varian real‐time position‐management system (Varian Medical Systems, Palo Alto, CA, USA). The scan conditions were as follows: gantry rotation time, 1.0 s; cine interval, 0.5 s; beam collimation, 40 mm; and slice thickness, 2.5 mm. The median (interquartile range) DLP and CTDI_vol_ were 503 mGy·cm (242–1,006 mGy·cm) and 62.9 mGy (53.9–69.6 mGy), respectively. The raw 4D‐CT images and corresponding respiratory signal data were transferred to a workstation and reconstructed according to 10 respiratory phase‐based bins of three‐dimensional CT images using Advantage 4D software (GE Healthcare). The patients were immobilized using the BodyFix device (Elekta AB, Stockholm, Sweden) and a T‐shaped holding bar. Abdominal respiratory motion was suppressed by covering with a plastic sheet with a pressure of 50–60 mbar, and oxygen was administered at a rate of 2 L/min via a nasal tube during the 4D‐CT scan. The scans were performed while breathing, with audio coaching for Patient #9 and without a plastic sheet for Patient #10.

#### 2.4.3 Image and data analysis

Tumor motions were tracked in each video of the AP and Lat projections, and the amplitudes of tumor motion were calculated in the horizontal and vertical directions, that is, the left‐right (LR) and superior‐inferior (SI) directions in the AP projection, and AP and SI directions in the Lat projection, respectively. The amplitude of tumor motion for each patient in the SI direction was determined as the average of the amplitudes in the AP and Lat projections. In addition, the square root of the sum of squares (SRSS) of the amplitude in the SI, LR, and AP directions was calculated, as follows:

$$\mathrm{SRSS}=\sqrt{SI^{2}+LR^{2}+AP^{2}}.$$

Geometric magnification was corrected for each patient using the tumor to back‐skin distance (in the AP projection) and the tumor to bodyside‐skin distance (in the Lat projection), as measured on the most recent trans‐axial CT image.

The 4D‐CT images were analyzed using the Eclipse11.0.47 treatment‐planning system (Varian Medical Systems). Tumor contouring was performed in the exhale and inhale phases, and the distance between the centroids of each contour was measured in the *X*, *Y*, and *Z* directions (i.e., the amplitude of the LR, AP, and SI direction, respectively).

Spearman's rank correlation and the Wilcoxon signed‐rank exact test were performed using EZR^25^ to compare the amplitudes of tumor motion between DCR and 4D‐CT in each direction, as well as of the SRSS values.

## 3 RESULTS

### 3.1 Phantom study

**FIGURE 2:**
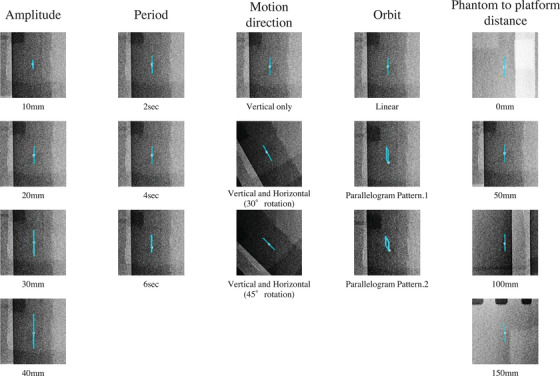
Static dynamic chest radiography (DCR) images of the phantom. The phantom motion pattern can be described in terms of the amplitude, period, motion direction, orbit, and phantom‐to‐platform distance. The trajectories of the phantom are denoted by blue colored lines (online version only)

Tracking of all motion patterns of the phantom was successful (Figure 2) (Videos [Link], [Link], [Link], [Link], [Link]). The absolute mean error for all motion patterns of the phantom was 0.60 mm (range: 0.06–1.53 mm) No pattern was clearly dominant in the overall mean error (Table 2).

**TABLE 2:** Absolute mean error for each phantom motion pattern. If the motion direction is diagonal, or the orbit is parallelogram, they are indicated by two lines, H and V in the table

Amplitude (mm)	Period (second)	Motion direction	Orbit	Phantom to platform distance (mm)	Mean (SD) error (mm)
10	4	V	Linear	100	0.63 (0.47)
20					0.33 (0.16)
30					1.30 (0.32)
40					1.17 (0.09)
20	2	V	Linear	100	0.17 (0.18)
	4				0.33 (0.16)
	6				0.76 (0.12)
20	4	V only	Linear	100	0.33 (0.16)
17.3		Diagonal	H		0.19 (0.18)
10.0			V		1.21 (0.07)
14.1		Diagonal	H		0.86 (0.04)
14.1			V		0.80 (0.00)
20	4	V only	Linear	100	0.33 (0.16)
3.0		H	Parallelogram		0.24 (0.20)
17.0		V			0.33 (0.05)
6.0		H	Parallelogram		0.53 (0.30)
14.0		V			0.39 (0.32)
20	4	V	Linear	0	0.50 (0.17)
				50	0.49 (0.01)
				100	0.33 (0.16)
				150	0.34 (0.33)

Abbreviations: H, motion in the horizontal direction; SD, standard deviation; V, motion in the vertical direction.

### 3.2 Clinical study

Motion tracking was successful for 24 of 32 (75%) tumor images in 12 AP and 10 Lat projection DCR videos. In two AP and three Lat projections, motion tracking was not successful (i.e., 5 of 32 (16%) tumor images); thus, manual correction was required. In one AP and two Lat projections, motion tracking was not possible for 3 of 32 (9%) tumor images because the tumors could not be detected in the videos. In particular, the tumor of Patient #7 was not detectable in either projection.

**FIGURE 3:**
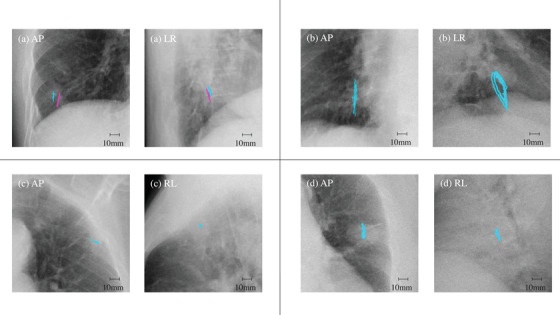
Enlarged static dynamic chest radiography (DCR) images of four patients in the anterior‐posterior (AP, left) and lateral (Lat, right) projections. The trajectories of the tumor are denoted by colored lines (online version only). LR, projection from left to right; RL, projection from right to left. (a) Right lower lung of Patient #1, which contained two tumors. The difference in length between the two colored (blue and pink) lines shows that the two tumors are located close to each other; however, the corresponding motion amplitudes do not coincide. The reason why the blue line is shorter than the pink line may be that the tumor corresponding to the blue line is attached to the chest wall (Video S6). (b) Right lower lung of Patient #9. The colored line in the LR projection denotes the hysteresis‐like motion of the tumor (Video S7). (c) Left upper lung of Patient #10. The colored line in the AP projection shows that the amplitude of tumor motion in the left‐right (LR) direction is larger than in the superior‐inferior (SI) direction (Video S8). (d) Left upper lung of Patient #14. The colored line in the AP projection shows the trajectory of the tumor, synchronized with the cardiac pulse. (Video S9)

**FIGURE 4:**
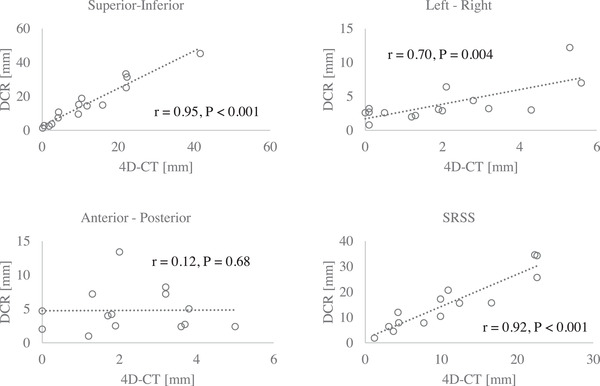
Correlations between the tumor motion amplitude in dynamic chest radiography (DCR) and respiratory‐gated four‐dimensional computed tomography (4D‐CT), in the superior‐inferior (SI), left‐right (LR), and anterior‐posterior (AP) directions, and between the square root of the sum of squares (SRSS) values. The sample sizes were based on the number of tumors detected by DCR

The tumors in the Lat projections of three patients showed hysteresis‐like motions, and in the AP projection of two patients, tumor motion was synchronized with the cardiac pulse (Figure 3) (Videos [Link], [Link], [Link], [Link]). The amplitudes of 15 of 16 (94%) tumor motions in the SI and LR directions, and 14 of 16 (88%) tumor motions in the AP direction, were measurable in DCR images. The motion amplitudes for DCR were significantly correlated with those for 4D‐CT in the SI and LR directions. The SRSS values were also significantly corelated (*r* = 0.95, *p* < 0.001; *r* = 0.70, *p* = 0.004, and *r* = 0.92, *p* < 0.001, respectively). However, the correlation was not significant in the AP direction (*r* = 0.12, *p* = 0.68) (Figure 4).

**FIGURE 5:**
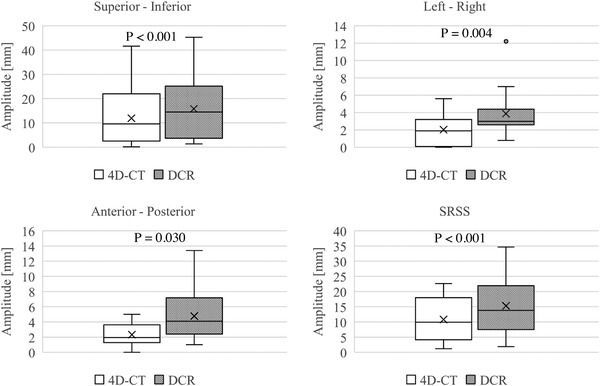
Comparison between the tumor motion amplitude in respiratory‐gated 4D‐CT and dynamic chest radiography (DCR) in the superior‐inferior (SI), left‐right (LR), and anterior‐posterior (AP) directions, and between the square root of the sum of squares (SRSS) values. The sample sizes were based on by the number of tumors detected by DCR

The median (interquartile range) amplitudes of tumor motion for DCR and 4D‐CT in the SI, LR, and AP directions were 14.5 mm (3.7–25.2 mm) versus 9.6 mm (2.5–22.0 mm); 3.0 mm (2.6–4.4 mm) versus 1.9 mm (0.1–3.2 mm); 4.1 mm (2.4–7.2 mm) versus 2.0 mm (1.3–3.6 mm), respectively. The SRSS values were 13.8 mm (7.5–21.9 mm) and 9.9 mm (4.1–18.0 mm). The motion amplitudes were significantly higher for DCR than for 4D‐CT in all (SI, LR, and AP) directions, as were the SRSS values (*p* < 0.001, *p* = 0.004, 0.030 and *p* < 0.001, respectively) (Figure 5).

## 4 DISCUSSION

Phantom and clinical studies were performed to determine the usefulness of our method, based on DCR and free analysis software (Kinovea), for assessing lung tumor motion. In the phantom study, the motion of the phantom was accurately determined, quantitatively and visually, in the DCR images. In the clinical study, the amplitudes of tumor motion for DCR showed good correlations with those for 4D‐CT, and the trajectories of most tumors were visualized automatically.

In the phantom study, the mean error of the amplitudes of phantom motion for DCR was less than 1 mm, which we consider to be within the tolerable range for clinical use. In addition, the amplitudes of the two‐dimensional motions (vertical and horizontal) were measured with high accuracy; this indicates the possibility of assessing three‐dimensional motion using two orthogonal projection images.

The results of the clinical study showed that the amplitudes of tumor motion for DCR were significantly correlated with those for 4D‐CT in the SI and LR directions, as were the SRSS values, but this was not the case for the AP direction. Hence, for DCR, the amplitude of tumor motion and the SRSS values were much more reliable in the SI and LR directions than in the AP direction. However, the motion amplitude in the SI direction is the most predominant and important parameter when determining the optimal treatment strategy.

**FIGURE 6:**
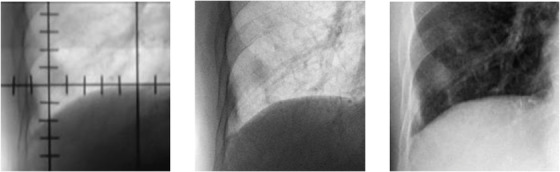
Fluoroscopy image (left), inverted dynamic chest radiography (DCR) image (center), and normal DCR image (right) of Patient #1

DCR solves the problems associated with fluoroscopy using an X‐ray simulator. First, the image quality of DCR is superior. Figure 6 shows fluoroscopic (Acuity; Varian Medical Systems) and DCR (normal and inverted) images of Patient #1. Halation and the crossline scale reduce tumor visibility in the fluoroscopic images, but they do not affect the DCR images. Both the DCR and X‐ray simulator use the same scintillator (CsI), but DCR appears to use higher‐quality FPDs (thus, it has higher DQEs).^26^ Compared with fluoroscopy, DCR uses the more sophisticated image‐processing techniques used in general radiography, including dynamic‐range compression and more extensive frequency enhancement.^27^ In the fluoroscopic study by McNair et al., 14 of 16 (82%) and 11 of 16 (65%) tumors were detectable in the AP and Lat projections, respectively.^28^ In the present study, 15 of 16 (94%) and 14 of 16 (88%) tumors were detectable in the AP and Lat DCR projections, respectively. The high image quality of DCR facilitates detection and tracking of both simple sinusoidal tumor motion and complex motions. Hysteresis‐like tumor motion, as well as motion synchronized with the cardiac pulse, was observed in the DCR images; these findings are consistent with several previous studies that used fluoroscopy, CT, and magnetic resonance imaging.^29^, ^30^, ^31^, ^32^, ^33^

Second, conventional fluoroscopic (i.e., visual) assessment of respiratory mobility lacks objectivity and quantitativity; our method may solve these problems. Software is used to analyze the DCR images, thus allowing several operators to repeatedly measure tumor motion and derive quantitative values that are more detailed than visual assessments.

DCR complements some disadvantages of 4D‐CT. The advantage of DCR over 4D‐CT is its robustness against breathing irregularities, which produce motion artifacts and geometric uncertainties in 4D‐CT scans.^5^, ^34^, ^35^ In DCR images, motion artifacts are not apparent, even under irregular breathing, due to the short image‐acquisition time (4.0/8.0 ms per image in the AP and Lat projections) and high temporal resolution (15 frames/s).

**FIGURE 7:**
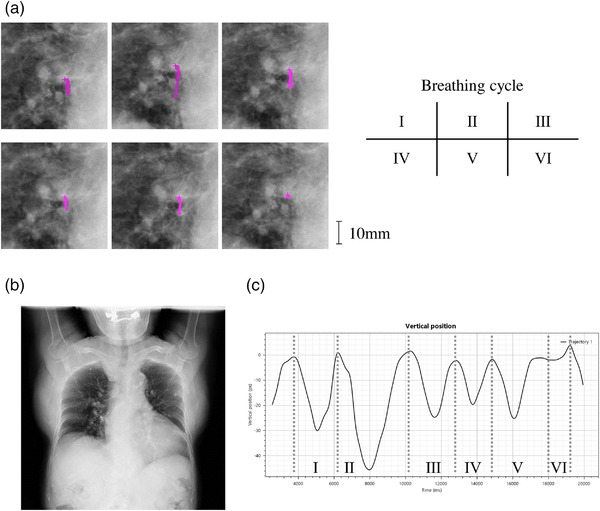
Breathing irregularity of Patient #4 revealed by a dynamic chest radiography (DCR) image. (a) Enlarged static DCR images of hilus of the right lung. The colored (pink: online version only) lines show the trajectory of the tumor in each breathing phase (I–VI) in the DCR image. (b) Static DCR image of the whole lung. (c) Graph exported from Kinovea. The vertical line shows the tumor position (pixel) in the superior‐anterior direction and the horizontal one shows the time (ms). In the six breathing cycles (I–VI), the periods and amplitudes of tumor motion during one cycle ranged from 1.6 to 4.2 s and 1.5 to 17.6 mm, respectively

DCR reveals patient‐specific respiratory patterns. The AAPM recommends observation of respiratory pattern changes over several breathing cycles.^1^ The DCR acquisition time of 20 s covers 4–7 cycles in older adults^36^; changes in respiratory patterns are apparent. This is an important advantage of DCR, compared to 4D‐CT. In a clinical study using Calypso beacons, Steiner et al. showed that respiratory motion was underpredicted by 4D‐CT because only one breathing cycle was used for measurement.^37^ Sarker et al. simulated virtual 4D‐CT using 155 normalized respiratory waveforms; they reported that the mean standard deviations in tumor positions during exhalation and inhalation varied by 1.7 and 3.0 mm, respectively.^34^ With DCR, the amplitude of tumor motion is measured as the difference in tumor position between the peak exhalation and peak inhalation during acquisition; thus, it includes deviations over several breathing cycles. As such, motion amplitudes are larger in DCR than in 4D‐CT, as demonstrated in this study. For DCR, extreme deviation due to irregular breathing is easy to identify as an outlier, as the respiratory pattern can be visualized. Figure 7 and Video S10 show the irregular breathing patterns of Patient #4 observed in cycles II and VI during DCR.

DCR is also cost‐effective. Fluoroscopic equipment (e.g., X‐ray simulators) is often unavailable in radiotherapy departments because of economic constraints and space limitations. A DCR system is composed of a ceiling‐mounted X‐ray tube, Bucky stand, Bucky table, and large field‐of‐view FPDs, all within a small space. Therefore, a DCR system can be used for dynamic imaging in radiotherapy, as well as general radiography. Radiotherapy departments with limited space or economic resources, precluding the installation of motion‐assessment systems, could set up a DCR system for general radiography. In addition, the Kinovea analysis software used by our method is freely available. Tsuchiya et al. and Tanaka et al. used in‐house software to measure the amplitude of tumor motion in DCR images.^38^, ^39^ However, due to the availability of Kinovea, there is no need to develop in‐house software to measure amplitude with DCR.

DCR also requires a lower radiation dose. The total surface dose used for DCR imaging (of the AP and Lat projections) in this study was approximately 11.4 mGy; this was lower than the total surface dose of general lumbar radiography (AP and Lat projections; 12.5 mGy according to the diagnostic reference level for Japan [2020] and 70 mGy according to the diagnostic reference level of the International Atomic Energy Agency [1996]).^40^, ^41^ In addition, DCR is rapid and requires only one technician, similar to conventional radiography. No respiratory monitoring is required, and DCR is not complex; technicians do not require specialist training. The entire procedure requires only 10–15 min, thus permitting high throughput in clinical settings. DCR aids early determination of the treatment strategy for SBRT; it is a simple, patient‐specific assessment for both high‐precision radiotherapy and conventional radiotherapy. With DCR, it is easy to assess the motions of multiple targets simultaneously in cases of advanced lung cancer. In addition, various auxiliary assessments, such as tumor motion under breath‐holding or the beam‐on time for respiratory gating radiotherapy, can be performed using DCR.

Thus, DCR could replace fluoroscopy using an X‐ray simulator. We are currently considering omission of fluoroscopy when DCR is performed.

Our method has several limitations. First, tumors with very low contrast (such as GGO), and those overlapping with other organs/structures (such as the heart, aorta, liver, or spine) in the DCR image, cannot be detected and assessed. In a previous Japanese study, about 27% (24/88) of early stage nonsmall‐cell lung cancers treated with SBRT had GGO.^24^ In such cases, assessment of the motion of structures close to the tumors (such as the diaphragm and vessels) may provide useful information. Second, the maximum 20‐s acquisition time of DCR is rather short for clinical use. The AAPM recommends a minimum of 30 s of digitally recorded imaging data, in conjunction with the respiration trace for respiratory gating methods.^1^ Third, tumor tracking by Kinovea could be improved. For example, Kinovea may lose an asymmetrically extended object that rotates. Finally, although Kinovea is free, users do not have access to technical support, and accuracy is not guaranteed.

The limitations of this study are that we did not include any patient with GGO, and the number of patients was small. When the number of patients increases, in future, it may be possible to analyze (for example) differences in the amplitudes and variations of trajectories depending on tumor localization in the lung lobes. Unlike in a previous study using implant markers,^29^ such analyses will be relatively simple and minimally invasive. Also, the breathing conditions during image acquisition differed between DCR and 4D‐CT; DCR was performed under free breathing, while 4D‐CT was performed under conditions wherein abdominal respiratory motion was suppressed by the BodyFix device. A previous study showed a reduction of at least 3 mm in tumor motion amplitude in the SI direction in 27% of patients when using the BodyFix device.^42^ The DCR imaging in this study was performed before patient‐specific immobilization devices were molded, and it was not possible to conduct the DCR and 4D‐CT parts of the study under the same breathing conditions. Therefore, the amplitudes of tumor motion were significantly larger for DCR than for 4D‐CT.

## 5 CONCLUSION

We developed a new method based on DCR and free analysis software for assessing lung tumor motion, then evaluated the usefulness of our approach. DCR has several advantages over conventional assessment methods, particularly fluoroscopy using an X‐ray simulator, and is a promising modality for assessing lung tumor motion in radiotherapy.

## AUTHOR CONTRIBUTIONS

All authors made substantial contributions to the conception and design of the study, data acquisition, data analysis, and interpretation of the data, and drafting and critical revision of the article for important intellectual content. All authors approved the final version of the manuscript for submission.

## CONFLICT OF INTEREST

The authors received research grants from Konica Minolta, Inc.

## Supporting information

Video S1. Dynamic chest radiography (DCR) images of the phantom, which exhibited motion patterns varying in amplitude. The trajectories of the phantom are denoted by blue lines.

Video S2. DCR images of various motion periods of the phantom. The trajectories of the phantom are denoted by blue lines.

Video S3. DCR images of various motion directions of the phantom. The trajectories of the phantom are denoted by blue lines.

Video S4. DCR images of phantom motion according to orbit. The trajectories of the phantom are denoted by blue lines.

Video S5. DCR images of phantom motion according to the distance from the phantom to the platform. The trajectories of the phantom are denoted by blue lines.

Video S6. DCR image of Patient #1 in the anterior‐posterior (AP) and lateral (Lat) projections. The trajectories of the two tumors are denoted by blue and pink lines.

Video S7. DCR image of Patient #9 in the AP and Lat projections. The trajectory of the tumor is denoted by the blue line.

Video S8. DCR image of Patient #10 in the AP and Lat projections. The trajectory of the tumor motion is denoted by the blue line.

Video S9. DCR image of Patient #14 in the AP and Lat projections. The trajectory of the tumor motion is denoted by the blue line.

Video S10. (a) DCR image of the entire lung of Patient #4. (b) Enlarged DCR images showing the trajectories of the tumor of Patient #4 in each breathing phase.
